# The Beneficial Effects of Chinese Herbal Monomers on Ameliorating Diabetic Cardiomyopathy via Nrf2 Signaling

**DOI:** 10.1155/2022/3959390

**Published:** 2022-05-24

**Authors:** Yiwei Gao, Wu Liu, Xin Su, Xinyi Li, Fangning Yu, Ning Zhang

**Affiliations:** ^1^Department of Nephrology and Endocrinology, Wangjing Hospital of China Academy of Chinese Medical Sciences, Beijing 100102, China; ^2^Guang'anmen Hospital of China Academy of Chinese Medical Sciences, Beijing 100053, China; ^3^Graduate School of Beijing University of Chinese Medicine, Beijing 100029, China

## Abstract

Diabetic cardiomyopathy (DCM) is the main factor responsible for poor prognosis and survival in patients with diabetes. The highly complex pathogenesis of DCM involves multiple signaling pathways, including nuclear factor-*κ*B (NF-*κ*B) signaling pathway, adenosine monophosphate-activated protein kinase (AMPK) signaling pathway, phosphatidylinositol 3-kinase-protein kinase B (Akt) signaling pathway, mitogen-activated protein kinase (MAPK) signaling pathway, and transforming growth factor-*β* (TGF-*β*) signaling pathway. Nuclear factor erythroid-2-related factor 2 (Nrf2) seems essential to the amelioration of the progression of DCM, not only through counterbalancing oxidative stress, but also through interacting with other signaling pathways to combat inflammation, the disorder in energy homeostasis and insulin signaling, and fibrosis. It has been evidenced that Chinese herbal monomers could attenuate DCM through the crosstalk of Nrf2 with other signaling pathways. This article has summarized the pathogenesis of DCM (especially in oxidative stress), the beneficial effects of ameliorating DCM via the Nrf2 signaling pathway and its crosstalk, and examples of Chinese herbal monomers. It will facilitate pharmacological research and development to promote the utilization of traditional Chinese medicine in DCM.

## 1. Introduction

Diabetic cardiomyopathy (DCM) is a cardiac muscle-specific microvascular complication, which progresses in individuals with diabetes mellitus (DM) but without other cardiac risk factors including coronary artery disease, hypertension, and significant valvular disease [1]. The Framingham Heart Study showed that over the past 50 years, the proportion of diabetes-caused cardiovascular diseases has increased, which emphasizes the need to pay more attention to the cardiac condition in patients with DM [2]. DCM, which elevates mortality in type 1 and type 2 diabetes mellitus (T1DM and T2DM) patients, leads to a poor prognosis, and individuals with DM were 2.3 times more likely to develop heart failure than those without [3, 4]. Around 22% of patients with T2DM develop heart failure [5]. Furthermore, recent studies showed that even in mildly elevated blood glucose (prediabetes), the risk of heart failure was increased and associated with a poor prognosis [6, 7]. To date, there is no special effective medicine for DCM [8]. However, a plethora of scientific evidence revealed that Chinese herbal monomers might be potential drugs for the treatment of DCM.

Nuclear factor erythroid-2-related factor 2 (Nrf2) is a potent antioxidant gene, which can regulate cell signaling, transcription, anabolic metabolism, and extracellular matrix (ECM) remodeling through jointly acting on multiple proteins [9]. Nrf2 activates comprehensive cellular defense processes by affecting nearly 500 genes, thus augmenting the whole ability of cells to perform redox balancing factors, detoxifying enzymes, stress response proteins, and metabolic enzymes [10, 11]. Increasing investigations have suggested that Nrf2 could ameliorate DCM via crosstalk with different signaling pathways, and some Chinese herbal monomers have proved to have the capability of prompting that mechanism. This review provides a contemporary view of the pathogenesis of DCM (especially in oxidative stress), the beneficial effects of ameliorating DCM via the Nrf2 signaling pathway and its crosstalk, and the examples of Chinese herbal monomers.

## 2. Review

### 2.1. Oxidative Stress in the Pathogenesis of DCM

The progression from DM to DCM is extremely complicated. The heart is one of the important target organs of diabetes. Under physiological conditions, insulin stimulates the uptake of glucose into the cardiac muscle to maintain glucose homeostasis; however, insulin insistence (IR) and hyperinsulinemia are associated with the metabolic disorder in cardiovascular diseases [12]. In patients with DM, the main abnormalities of the inner environment are hyperglycemia, systemic IR, and hyperinsulinemia [1]. In those conditions, these abnormalities instigate disorders of systemic metabolism, activation of the sympathetic nervous system and renin-angiotensin-aldosterone system, response for maladaptive immune, inflammation, and accretion of advanced glycation end products (AGEs) that further prompt oxidative stress and lipid accumulation [13, 14]. Oxidative stress could trigger mitochondrial dysfunction, and endoplasmic reticulum stress (ERS), impair calcium handling, and increase Ca^2+^ sensitivity and Ca^2+^ influx [13]. The imbalance between mitophagy and mitochondrial biogenesis leads to damage to cardiomyocytes and fewer supply of energy to the myocardia [13, 15, 16]. Oxidative stress and ERS could induce abnormalities of calcium handling, which lead to diastolic dysfunction [13]. Besides, the interaction of reactive oxygen species (ROS), dysfunction mitochondrial, ERS, and abnormal calcium handling ultimately causes apoptosis [13]. Apoptosis is considered a major mechanism in maintaining cellular homeostasis in general, and it plays a crucial role in normal tissue turnover, immune development, and defense [17]. However, an increased level of apoptosis causes excess cell death in many diseases [17]. Regardless of the diabetes type, highly conserved intracellular pathways of apoptosis are triggered and lead to a point of no return in apoptosis to influence *β*-cells, which provokes more metabolic dysfunctions and thereby cause diabetic complications [18]. The results of the highly complex interaction of multiple distinct but overlapping mechanisms are some typical changes in the structure of the heart, including cardiac stiffness, hypertrophy, and fibrosis, leading to cardiac dysfunction, combined with cardiomyocyte death that will promote the progress of heart failure [1, 13].

Behind these pathological features, there are interactions among multiple signaling pathways. ROS could enhance nuclear factor-*κ*B (NF-*κ*B) signaling pathway as a maladaptive immune modulation that prompts cardiac remodeling and fibrosis [12, 19]. The increased ROS and impaired adenosine monophosphate-activated protein kinase (AMPK) signaling pathway further decrease fatty acid oxidation (FAO) and then lead to lipid accumulation and diastolic dysfunction [13]. Inappropriate activation of the renin-angiotensin-aldosterone system impairs the phosphatidylinositol 3-kinase (PI3K)-protein kinase B (Akt) signaling pathway, further increasing intracellular Ca^2+^ levels and Ca^2+^ sensitivity and then resulting in cardiac fibrosis/stiffness and diastolic dysfunction [12]. AGEs could increase fibrosis, cardiac stiffness, and impaired diastolic relaxation by increasing the production of ROS and activating the mitogen-activated protein kinase (MAPK) signaling pathway [13]. Meanwhile, AGEs could stimulate the expression of collagen, the crosslinks of collagen molecules, and the accumulation of collagen [13, 20, 21]. The receptor for AGEs could induce the expression of transforming growth factor-*β* (TGF-*β*) to elicit the forming of myofibroblasts [22, 23].

Oxidative stress, an imbalance status between prooxidants and antioxidants, may perform a central role in the pathogenesis of DCM via impacting *β*-cells and cardiac cells. A constant weakening in *β*-cells quantities and utility is one of the characteristics of the natural history of diabetes, which tightly relates to microvascular or macrovascular complications of DM, including DCM [24]. The pancreatic *β*-cells, with lower levels of free radical detoxifying and redox-regulating enzymes, contrasted to other cell types, may be at a higher hazard for oxidative injury with boosted sensitivity for apoptosis [25]. ROS and reactive nitrogen species (RNS) are the two chief cellular generation sites of redox-reactive species [19]. Under physiological conditions, ROS/RNS regulates insulin secretion and insulin action; conversely, under pathological conditions, ROS/RNS prompts the deactivation of metabolic enzymes, suppression of insulin secretion, and death of *β*-cells [26]. The activity of nicotinamide adenine dinucleotide phosphate [NAD(P)H] oxidase (NOX) elevated upon Ca^2+^ stimulation, then prompt ROS accumulation in *β*-cells increased rapidly, and attain harmful levels to influence the progression of DM and its complications [25]. NOXs, an important ROS-producing enzyme, not only trigger oxidative damage in *β*-cells but also regulate both adaptive and maladaptive changes in the cardiomyocytes [27]. The physiological amounts of nitric oxide (NO) are a vital coupling factor in insulin-secreting cells; however, the excessive NO production, which may relate to inflammation, can generate oxidative/nitrosative stress, which is one of the crucial procedures of *β*-cells death [19, 28].

ROS is regarded as a prototypical senescence inducer, and the considerable amount of ROS and RNS in adult cardiac muscle cells leads to cardiotoxicity [29]. ROS-induced aging and cardiotoxicity prompt cardiac stem cells senescence and then reduce cardiac muscle function, especially in patients with DM [29]. Besides broad oxidation inducing cell dysfunction, necrosis, or apoptosis, dysregulated ROS/RNS signaling also leads to specific posttranslational modifications which could alter the function of vital cellular proteins and signaling pathways in the heart [30]. For example, bromodomain-containing protein 4 is a critical protein in the modulation of various biological processes, and its expression has been detected upregulated in the DCM [31, 32]. ROS production could trigger the expression of bromodomain-containing protein 4 to prompt cardiac hypertrophy, and this progression also relates to signaling pathways for inflammation, fibrosis, and so on [31]. NO is an oxygen-derived free radical and is synthesized by three NO synthase isoforms including inducible NO synthase (iNOS), endothelial NOS (eNOS), and neuronal nitric oxide synthase [19, 33, 34]. Abnormalities in vascular NO production and transport accompany many disease states, including cardiovascular diseases and diabetes [34]. The pathological amounts of NO are related to vascular endothelial dysfunction, which is considered a major mediator in diabetic cardiomyopathy [35]. The reduction of bioavailability of NO in the vasculature is one of the characteristics of IR, and improving the bioavailability of NO could also help coronary vasodilation [36, 37].

**Figure 1:**
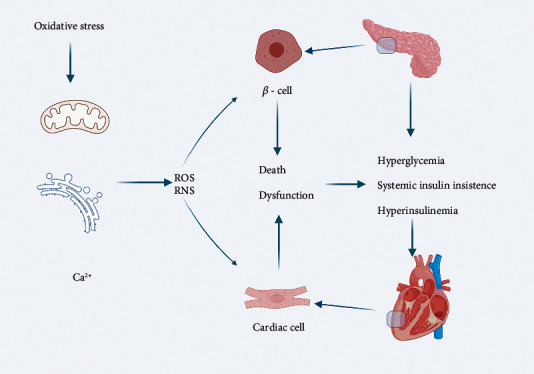
Oxidative stress in the pathogenesis of DCM (created with BioRender.com). In patients with diabetes, the main abnormalities of the inner environment are hyperglycemia, systemic insulin insistence, and hyperinsulinemia. Oxidative stress could trigger mitochondrial dysfunction and endoplasmic reticulum stress, and impair calcium handling and increase Ca^2+^ sensitivity and Ca^2+^ influx. ROS/RNS could impact both *β*-cells and cardiac cells leading to cell death and dysfunction. ROS: reactive oxygen species; RNS: reactive nitrogen species.

The pathogenesis of DCM is extremely complex and involves multiple signaling pathways. Oxidative stress is the major factor responsible for poor prognosis and survival in patients with DCM, and it could impact both *β*-cells and cardiac cells. The mechanisms of oxidative stress in DCM have been summarized in Figure 1. Antioxidation is one of the promising therapeutic strategies for DCM and calls for more attention.

### 2.2. Relationship of Nrf2 and DCM

Nrf2 performs a critical character in counterbalancing oxidative stress and inflammation. Kelch-like ECH-associated protein 1 (Keap1) sequesters Nrf2 in cytoplasmic usually, but under conditions of oxidative stress, it resolves with Nrf2 in a dose-independent manner [38]. The resolved newly synthesized Nrf2 translocates into the nucleus and then binds to the small Maf proteins to form a new protein dimer [10]. Furthermore, the heterodimer can recognize the antioxidant response elements (AREs), which locate in the regulatory domains of multiple defense enzyme genes [11]. AREs then transcript heme oxygenase-1 (HO-1), NAD(P)H quinone dehydrogenase-1 (NQO1), superoxide dismutase (SOD), catalase (CAT), glutathione peroxidase (GPX), glutathione S-transferase (GST), and *γ*-glutamylcysteine synthetase (*γ*-GCS) to defend the cell against oxidative stress [39, 40].

Nrf2 is broadly accepted as having a remarkable role in combating oxidative stress, and investigations have claimed that the expression of Nrf2 in diabetic animals and patients is significantly diminished [41, 42]. The decreased expression of Nrf2 leads to cardiac damage and is correlated to IR, abnormal angiogenesis, and endothelial dysfunction [41]. However, increasing the expression of Nrf2 can guard the cardiac cells and heart against the hyperglycemia environment *in vitro* and *in vivo* [42]. Chinese herbal monomers could upregulate Nrf2 to reverse this condition. Abdelsamia et al. suggested the advantages of metformin/curcumin combination in counteracting DCM [43]. They treated diabetic rats with curcumin (100 mg/kg/d) for 6 weeks and then observed Nrf2; HO-1 upregulated; and the metformin/curcumin combination group is superior to the metformin and curcumin group [43]. Atta et al. noted that 12 weeks oral of thymoquinone (50 mg/kg/d) in diabetic rats could upregulate Nrf2 and SOD, as well as downregulate iNOS and NO [44].

Previous studies also suggested that activating Nrf2/HO-1 pathway could positively attenuate the death of cardiomyocytes [45]. The strategy of targeting Nrf2 could enhance the expression of Nrf2 in the cardiac to elevate the expression of HO-1 in the myocardial and then diminish cardiac hypertrophy and cardiac dysfunction [46]. Wang et al. fed T1DM mice with resveratrol (10 mg/kg per day) for 1 month and afterward observed for 6 months and showed that cardiac function improved and fibrosis reduced which is accompanied by upregulating Nrf2, HO-1, SOD, and NQO1 [47]. Dong et al. evidenced in vitro that gastrodin could defend against hyperglycemia-induced cardiomyocyte toxicity through upregulating Nrf2, SOD, and CAT [48]. Duan et al. found that *Aralia taibaiensis* could reduce intracellular ROS levels and cell oxidative injury accompanied by enhancing the expression of Nrf2, SOD, and GSH [49].

The Nrf2 system is also responsible for maintaining lipid metabolism and glucose metabolism by regulating glucose utilization and insulin secretion to convert the progression of DM [50]. Castillo et al. treated rats with quercetin (0.5% w/w) for 4 weeks and then observed quercetin counteracted hyperglycemia-induced bioenergetic deterioration, including avoiding cardiac cholesterol accumulation, accompanied by upregulating Nrf2, HO-1, SOD, and proliferator-activated receptor gamma coactivator 1-alpha (PGC-1*α*) [51]. PGC-1*α* is regarded as a master regulator of mitochondria, which is related to intracellular energy homeostasis [52]. In a word, Nrf2, as an antioxidant factor, could efficiently ameliorate DCM.

### 2.3. Main Signaling Pathways in the Pathogenesis of DCM and Crosstalk with Nrf2

The pathogenesis of DCM involves diverse signaling pathways to exercise different functions, and these mechanisms are summarized in Table 1. For ameliorating DCM, Nrf2 plays a pivotal role in crosstalk with these pathways and that would be discussed in detail in the following text.

#### 2.3.1. Effects of NF-*κ*B Signaling Pathway and Crosstalk with Nrf2

NF-*κ*B is one of the major signaling pathways involved in the pathogenesis of DCM. Mainly transcription factors in mammals of the NF-*κ*B family include p50, p52, p65, Rel, and RelB [53, 54]. NF-*κ*B is expressed in nearly all cell sorts, and the family of inhibitors of NF-*κ*B (I*κ*B) could make it inactive in the cytoplasm [55, 56]. This signaling pathway could be activated in multiple ways, including ROS and RNS levels, toll-like receptors (TLRs), interleukin-1 (IL-1), IL-6, and tumor necrosis factor *α* (TNF-*α*) [19, 55, 57, 58, 59]. TLRs, inside the human body, it is named TLR4, are components of the innate immune, whose activation can produce inflammatory cytokines and systematically affects vascular function and remodeling [60]. Myeloid differentiation primary response protein 88 (MyD88), one of the adapters of TLR4, is a kinase that performs an essential role in triggering NF-*κ*B signaling [57]. TLR4 can also bind to NOX4 and subsequently generate ROS, thus provoking oxidative damage [19, 61]. Proinflammatory cytokines, such as IL-1*β*, are produced by inflammasomes, which are a group of protein complexes built around several proteins, including nucleotide-binding oligomerization domain-like receptor protein 3 (NLRP3) [62]. Besides that, NF-*κ*B could transcript some cell adhesion molecules, which firmly adhesion to leukocytes to migrate into injured tissues, such as intercellular adhesion molecule-1 (ICAM-1) and vascular cell adhesion molecular-1 (VCAM-1) [63]. Monocyte chemoattractant protein-1 (MCP-1), a member of chemotactic cytokines, could promote adhesion via the upregulated related receptor, such as the receptor of ICAM-1 [64].

NF-*κ*B signaling pathway performs a vital part in the pathophysiology of DCM through involving in the transcription of different proinflammatory and inflammatory [65]. During inflammatory responses, cellular events are tightly associated with redox balance [66]. Between Nrf2 and NF-*κ*B signaling pathways, there is an existing complex and dynamic interplay, and both modulate the physiological homeostasis of cellular redox status and responses to stress and inflammation [10]. Nrf2 signaling pathway could decrease the production of ROS in intracellular and then could inhibit proinflammatory signals in general [67]. Nrf2 plays a major role in anti-inflammatory and includes counteracting NF-*κ*B-driven inflammatory, and this supposedly has been evidenced by multiple studies [10, 68]. Raish et al. stated that sinapic acid (20 and 40 mg/kg oral for 12 weeks) can upregulate GPX, SOD, CAT, I*κ*B-*α*/*β*, Nrf2, and HO-1 while downregulating TNF-*α*, IL-6, and NF-*κ*B [45]. Lian et al. observed the potential of chrysophanol for antioxidant and anti-inflammation is Nrf2-dependent [69]. They gave mice 25 and 50 mg/kg/d chrysophanol solution for 19 weeks and then noted Nrf2, HO-1 are upregulated and is accompanied with IL-6, IL-18, IL-1*β*, TNF-*α*, ICAM-1, and VCAM-1 are downregulated, but this result does not show in Nrf2 knockout mice [69]. Chen et al. also observed this dependency; they found that kaempferol can enhance Nrf2 activity in cells and upregulate HO-1, NQO1, SOD, and I*κ*B-*α*, as well as downregulate TNF-*α* and IL-6, but when knockdown of Nrf2 in H9c2 cells, kaempferol has no attenuation effect on ROS production [70].

NF-*κ*B signaling pathway also performs a crucial character in apoptosis. In *β*-cells, activated NF-*κ*B could upregulate proapoptotic (e.g., Bax) and downregulate antiapoptotic (e.g., Bcl2) [19]. Besides, excessive production of ROS provokes the apoptosis of cells through combination with inflammatory [19]. Nrf2 signaling pathway could inhibit apoptosis in multiple ways, such as transcript antioxidant factors, suppressing NF-*κ*B signaling pathway. Liang's study could approve this result. After 12 weeks of oral for 1, 10, and 20 mg/kg/d in diabetes mice, andrographolide could upregulate SOD, Nrf2, HO-1, and I*κ*B-*α*, as well as downregulate p65, NF-*κ*B, TNF-*α*, IL-1*β*, IL-6, and Bax/Bc12 [71]. Li et al. found that piceatannol in vitro could enhance the expression of Bc12, Nrf2, HO-1, SOD, and I*κ*B-*α* and, meanwhile, reduce the expression of Bax, p65, and caspase3 [72]. The family of caspase is related to apoptosis *β*-cells [19].

**Figure 2:**
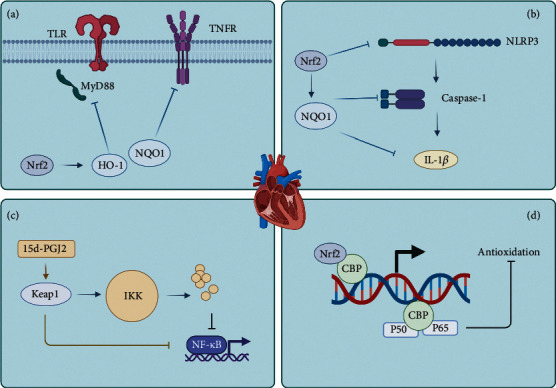
The crosstalk between Nrf2 and NF-*κ*B signaling pathway (created with BioRender.com). (a) Nrf2-dependent antioxidant genes could block TLR and TNF-dependent signaling. (b) Nrf2 and NQO1 inhibit the priming step of NLRP3, and suppress caspase-1 cleavage and IL-1*β* generation. (c) Keap1 could ubiquitinate IKK and be targeted by 15d-PGJ2. (d) Nrf2 and NF-*κ*B could compete to combine with CBP. Nrf2: nuclear factor erythroid-2 related factor 2; NF-*κ*B: nuclear factor-*κ*B; HO-1: heme oxygenase-1; NQO1: NAD(P)H quinone dehydrogenase-1; TLR: toll-like receptors; TNFR: tumor necrosis factor receptor; NLRP3: nucleotide-binding oligomerization domain-like receptor protein 3; IL-1*β*: interleukin-1*β*; Keap1: kelch-like ECH-associated protein 1; IKK: inhibitors of NF-*κ*B kinase; CBP: cAMP-response-element-binding protein-binding protein.

The main crosstalk mechanisms in Nrf2 and NF-*κ*B signaling pathway are as follows. Firstly, Nrf2-dependent antioxidant genes, such as HO-1 and NQO1, could limit the activation of NF-*κ*B to attenuate inflammation via blocking TNF and TLR4-depending signaling pathways [68, 73]. Yan et al. observed that scutellarin could regulate both Keap1/Nrf2/ARE and TLR4/MyD88/NF-*κ*B signaling pathways [74]. According to Yan's study, scutellarin could upregulate the expression of SOD, CAT, GPX, GST, Nrf2, NQO1, HO-1, and I*κ*B-*β* while downregulating Keap1, TLR4, Myd88, p50, IL-6, and TNF-*α* after a 6 weeks oral (10 or 20 mg/kg/day) [74]. Xu et al. gave mice bixin solution 50, 100, and 200 mg/kg/d for 14 weeks and then found that Nrf2, SOD, HO-1, and CAT upregulated; meanwhile, TLR4, Myd88, I*κ*B-*α*, and NF-*κ*B downregulated [75]. Enhancing the expression of HO-1 not only inhibits the TNF-dependent activation of NF-*κ*B but also reduces VCAM-1 expression in aortic endothelial cells; the behind mechanism may be that HO-1 can impede the transcriptional machinery of NF-*κ*B in the nucleus [76]. Li et al. observed that 15 weeks oral of luteolin (20 mg/kg/d) could upregulate Nrf2, HO-1, and NQO1; meanwhile, it could downregulate IL-1*β*, IL-6, TNF-*α*, MCP-1, ICAM, and VCAM [77]. Secondly, Nrf2 could impede the activation of NLRP3 inflammasome. Nrf2 and NQO1 are involved in the progression, impeding the priming step to decrease the activity of NLRP3 inflammasome, and it also suppresses caspase-1 cleavage and subsequent IL-1*β* generation [78]. Thirdly, Keap1 could inhibit the activity of NF-*κ*B via ubiquitinating I*κ*B kinase [79]. Furthermore, Keap1 could be targeted by 15d-PGJ2 to initiate gene transcription with an overall anti-inflammatory result [80]. 15d-PGJ2 is a product of NF-*κ*B-induced cyclooxygenase-2, where interesting is that NF-*κ*B system could manage its termination by expression of other target genes [80]. Fourthly, NF-*κ*B could compete with Nrf2 to combine with cAMP-response-element-binding protein-binding protein, a transcriptional co-activator [68]. The mechanisms have been summarized in the figure (see Figure 2). In conclusion, the NF-*κ*B signaling pathway triggering inflammation and apoptosis to lead the poor prognosis of DCM and Nrf2 could improve this via crosstalk with it.

#### 2.3.2. Effects of AMPK Signaling Pathway and Crosstalk with Nrf2

AMPK has been regarded as an enzyme that performs a crucial part in maintaining energy homeostasis, reduction of ROS production in the cytosol, and utilization of glucose [81–83]. The activity of AMPK was considerably decreased in DCM, and increasing the activity of AMPK would significantly diminish lipid accumulation and revamp cardiac function [8]. In cardiac, AMPK is a major kinase to regulate myocardial metabolism through controlling numerous metabolic pathways, such as lipid metabolism and utilization [8, 83]. Under normal physiological conditions, the adult heart gains about 50-75% of its acetyl coenzyme A (CoA)-derived ATP from FAO, but also could rapidly adjust to alterations in substrate availability for the generation of ATP to incessantly maintain its energy requirements, which termed “metabolic flexibility” [83]. However, in pathological cardiac hypertrophy and dilated cardiomyopathy, there would be some changes in transcription that prompt the diminish of this metabolic flexibility, which contributes to the pathogenesis of heart failure [83]. Once AMPK is activated, it could increase fatty acids entering the mitochondria through carnitine palmitoyl CoA transferase 1 for FAO [84]. Acetyl-CoA carboxylase (ACC) is a protein that could catalyze the transformation of acetyl CoA to malonyl-CoA, and malonyl-CoA could negatively regulate carnitine palmitoyl CoA transferase 1 [85]. Activated AMPK could diminish malonyl-CoA levels and increase FAO through phosphorylating and inhibiting ACC [86]. Furthermore, both AMPK and silent information regulator 1 (SIRT1) are regarded as the gatekeepers of the activity of PGC-1*α*, and the activated AMPK/SIRT1/PGC1-*α* signaling pathway contributes to a regulatory network for metabolic homeostasis [52].

Both AMPK and Nrf1/2 are the crucial regulator of mitochondrial dynamics and synergistic to maintain cardiovascular energy homeostasis [15, 87]. Enhancing the expression of Nrf2 could help AMPK to improve cardiac function. Li et al. stated that bailcalin improves diabetes-induced cardiac dysfunction via AMPK/Nrf2 signaling [8]. After administering diabetic mice with bailcalin (100 mg/kg/d, 4 months), it was found that it can upregulate AMPK*α*, CPT-1, PGC1-*α*, glutathione (GSH), SOD, and Nrf2 while downregulating atrial natriuretic peptide (ANP), B-type natriuretic peptide (BNP), *β*-myosin heavy chain (*β*-MHC), ACC, and oxidized glutathione (GSSG) [8]. GSH is regarded as one of the most essential scavengers of ROS, and its ratio with GSSG may be considered a biomarker of oxidative stress [88]. BNP is a valuable indicator in the diagnosis of heart failure, and its elevation is correlated with disease severity, especially left ventricular systolic ejection fraction and left ventricular diastolic function [89]. The meaning of ANP is nearly the same as BNP, but it has a different mechanism [90]. In cardiomyocytes, isoforms of *β*-MHC have been evidenced to change cardiac muscle function both in healthy developing and diseased hearts [91]. Both BNP and *β*-MHC are cardiac hypertrophy marker proteins [92]. Du et al. found that in vitro notoginsenoside R1 could upregulate Nrf2, HO-1, and AMPK, as well as downregulate ANP and BNP [93].

Besides, AMPK could prompt the Nrf2-mediated antioxidative cascade while inhibiting inflammation via suppression of TLR-mediated proinflammatory cascades [94]. Kosuru et al. stated that pterostilbene, 8 weeks oral for 20 mg/kg/d in rats, could upregulate SOD, CAT, GSH, GPX, PGC-1*α*, Nrf2, HO-1, and AMPK; meanwhile, it could downregulate IL-1*β*, IL-6, TNF-*α*, NF-*κ*B, TLR4, and NLRP3 [95]. Zhao et al. observed that fortunellin, 8 weeks oral for 10, 20, and 30 mg/kg in mice, could upregulate SOD, Nrf2, HO-1, and AMPK and downregulate TNF-*α*, IL-1*β*, IL-6, IL-18, NF-*κ*B, and Keap1 [96]. Altamimi et al. revealed that ellagic acid, 8 weeks for 100 mg/kg/d in rats, could upregulate GSH, SOD, Nrf2, and SIRT1, as well as downregulate BNP, TNF-*α*, and IL-6 [97].

Although whether Nrf2 is a direct molecular target of AMPK is unclear, Nrf2 could be activated in an AMPK-dependent way and as a downstream factor [94]. The crosstalk mechanisms between Nrf2 and AMPK signaling pathway are concluded in the following aspects. Firstly, glycogen synthase kinase 3*β* (GSK3*β*), which is regarded as an activation switch of Nrf2 gene expression, is a key protein in the crosstalk between Nrf2 and AMPK. Nrf2 could be phosphorylated by AMPK at the Ser550 residue, and then combine with AMPK-mediated GSK3*β* inhibition, further enhancing the nuclear accumulation of Nrf2 for ARE-driven gene transactivation [98]. Phosphorylated GSK3*β* was also found that this could improve cardiac function [99]. Cao et al. found in vitro that Z-ligustilide could restore cardiomyocyte dysfunction via upregulating AMPK, Nrf2, and SOD while downregulating GSK3*β* [100]. Secondly, activated AMPK could promote p62-dependent autophagic degradation of Keap1, which leads Nrf2 to separate from Keap1 and translocate to the nucleus [101]. Thirdly, SIRT1, which could be activated by AMPK via increasing the substrate, could regulate Nrf2 to attenuate oxidative damage [102]. Besides, a family of secreted frizzled-related proteins (Sfrps) recently had been reported to be widely associated with the pathogenesis and prognosis of DCM, including apoptosis, inflammation, and oxidative stress, and then lead to the events of cardiac fibrosis and even heart failure [103–108]. Although Sfrps has been evidenced could reduce oxidative stress in an AMPK/PGC1-*α*-dependent manner, studies are deficient about the direct relationship between Nrf2 and Sfrps [108]. However, between Nrf2 and Sfrps, there are so many same proteins involved, such as GSK3*β* and PGC1-*α*, and the related studies should be expected [104, 105, 108]. In short, besides combating oxidative stress, AMPK and Nrf2 jointly work to revamp cardiac function via maintaining heart energy homeostasis, improving utilization of glucose, and decreasing lipid accumulation and inflammation based on the above mechanisms.

#### 2.3.3. Effects of Akt Signaling Pathway, and Crosstalk with Nrf2

PI3K/Akt signaling pathway also significantly influences the prognosis of DCM. IR could independently predict the mortality of individuals with heart failure [109]. Akt signaling pathway is one typical pathway responsible for cellular insulin signaling, which is beneficial to the glucose uptake in the heart [13]. Impairment of the insulin-induced activation of PI3K/Akt is one of the characteristics of IR [109]. Activated Akt signaling pathway could ameliorate cardiac IR [110]. Recently, accumulating evidence has suggested that oxidative stress plays a causal role in the cardiac complications of IR too [109]. Akt signaling pathway evidenced that it could enhance the expression of Nrf2 and then jointly against IR to improve cardiac function [111, 112]. Xu et al. observed that cardiac function improved in mice after 4 weeks of intraperitoneal injection with scutellarin (5, 10, 20 mg/kg), accompanied by enhancing the expression of Nrf2, HO-1, and Akt [113].

Akt signaling pathway could be activated in multiple ways, including insulin receptor substrate (IRS), and estrogen receptors. IRS represented a classical insulin-induced way to activate Akt signaling pathway, which contributes to ameliorating prognosis, including improving cardiac function and apoptosis. Ma et al. evidenced that low expression of SIRT1 induces the decrease of IRS-2 and further does not activate Akt signaling pathway [110]. According to Ma's study, the level of ANP and BNP significantly increased in the SIRT1 knockout mice, and 5 consecutive days treated with resveratrol (25 mg/kg/d) in mice could reverse this condition, by enhancing the expression of Nrf2, SIRT1 [110]. Furthermore, IRS-1 could initiate eNOS through Akt activating way [37]. In diabetic rats, the blocked PI3K/Akt signaling pathway results in the reduction of protein expression of eNOS, which could also regulate the level of apoptosis [112]. Liu et al. showed that spiraeoside in vitro could upregulate Akt, Nrf2, HO-1, Bcl2, SOD, GPX, and CAT and downregulate caspase3, caspase7, and Bax [114]. Estrogen receptors-*α*36-G protein-coupled estrogen receptor signaling complex could rapidly induce the generating of ceramide, which is necessary for signaling of ceramide-protein kinase C *ζ*-casein kinase 2 (CK2) [115]. CK2 further supports the activation of diverse signaling kinases, including Akt signaling [115].

The crosstalk mechanisms between Akt and Nrf2 are the following aspects. Firstly, GSK3*β* is regarded as a crucial protein. GSK3*β*, a multifunctional serine/threonine kinase, could phosphorylate Fyn, and then phosphorylate Nrf2 tyrosine 568, finally provoking the degradation of Nrf2 [116]. Akt could phosphorylate GSK3*β* at Ser 9 to make it deactivate to facilitate the accumulation of Nrf2 [111, 116]. Zhang et al. found that in vitro myricitrin could increase Nrf2, HO-1, *γ*-GCS, NQO1, and Akt, as well as downregulate GSK3*β* [117]. Duan et al. showed that every other day for 15 days oral for 10, 20, and 40 mg/kg in diabetic mice, butin could upregulate SOD, Nrf2, HO-1, and Akt, as well as downregulate Keap1, GSK3*β*, and Fyn [116]. Besides, CK2 contributes to the accumulation of Nrf2 not only by activating the PI3K/Akt axis but also by directly phosphorylating Nrf2 to enhance its stability [115]. Briefly, Akt and Nrf2 signaling pathways could alleviate IR and apoptosis to improve the prognosis of DCM through regulating cellular insulin signaling.

#### 2.3.4. Effects of MAPK Signaling Pathway and Crosstalk with Nrf2

MAPK is a vital target signaling pathway for treating DCM. MAPK is a key signal transduction pathway in regulating cellular insulin signaling, which mainly relates to the disturbance in the metabolic and growth effects of insulin signaling [13]. The activated MAPK signaling pathway is related to growth and remodeling responses, which leads to myocardial hypertrophy, cardiac fibrosis, impaired myocardial endothelial signaling, and death of myocardial and endothelial cells [13]. Besides, MAPK is regarded canonical intracellular signaling pathway related to inflammation and immune [118]. Downregulating the MAPK pathway could alleviate chronic inflammation in diabetic mice via Chinese herbal monomers, such as berberine [119].

Chinese herbal monomers have been observed that they can regulate MAPK and Nrf2 signaling pathways in cardiac at the same time. MAPK family includes c-Jun N-terminal kinase (JNK), extracellular signal-regulated kinase (ERK), and p38. Overactivated phosphorylated ERK, which tightly relates to IR in cardiac, is always accompanied by a depressed expression of cardiac Nrf2 [109]. Furthermore, insulin-induced ERK activity was significantly decreased by the forced activation of Nrf2, which indicated that activation of Nrf2 could diminish the activity of oxidative stress-induced ERK in adult cardiomyocytes [109]. Chinese herbal monomers have been evidenced that they could inhibit ERK1/2 and p38 MAPK phosphorylation in angiotensin II-treated neonatal rat ventricular myocytes, which contribute to alleviating cardiac hypertrophy [120, 121]. Activated JNK, a key marker of tissue injury, was previously shown to relate to IR, increased ROS generation, and ERS under hyperglycemic conditions [122, 123]. The Nrf2 inhibitor could increase the expression of JNK [123]. The ERK1/2 and JNK are downstream factors of the Nrf2 pathway, involved in DCM, and upregulated by NOX and Nrf2 deficiency-stimulated ROS production [123]. Gu et al. showed isoliquiritigenin in vitro could upregulate Nrf2 and HO-1 and meanwhile downregulate TNF-*α*, IL-6, IL-1*β*, VCAM-1, MCP-1, JNK, ERK, and p38 [118]. Lu et al. suggested that hinokinin could protect against cardiac injury; they treated diabetic mice with hinokinin (20 and 40 mg/kg) for 6 weeks and then observed that Nrf2, HO-1, and SOD upregulated, while Keap1, JNK1, ERK1/2, and p38 downregulated [124]. Ni et al. found that in vitro salidroside could upregulate Nrf2 and HO-1, as well as downregulate ERK, JNK, and p38 to protect against cardiomyocyte apoptosis and ventricular remodeling [125]. Nrf2 could inhibit MAPK signaling pathway to ameliorate the prognosis of DCM; however, the relationship between Nrf2 and MAPK calls for more research details.

#### 2.3.5. Effects of TGF-*β* Signaling Pathway and Crosstalk with Nrf2

Fibrotic diseases are a result of an imbalance between profibrotic and antifibrotic cytokines and secreted proteins, whose character is excessive scarring caused by excessive production, deposition, and contraction of ECM [126]. The degradation of ECM is regulated by matrix metalloproteinases (MMPs), and the dysregulation of MMPs function, specifically MMP-2 and MMP-9, could provoke myocardial remodeling and the development of heart failure [127, 128]. Fibrosis is one of the most prevalent characteristics of diabetes, and recent evidence posted the term “redox fibrosis” [129]. The term “redox fibrosis” means that oxidative stress and the antioxidant system might be the essential mechanism behind fibrosis development and persistence, and the potential target of antifibrosis is the antioxidant system [129].

TGF-*β* and connective tissue growth factor (CTGF) are important profibrotic proteins [126]. TGF-*β* could induce fibroblasts to synthesize and contract ECM, and it has been regarded as a dominant regulator in the responding of fibrotic for a long time, which performs a central part of fibrogenesis in almost all organs [129, 130]. CTGF, whose regulation is mediated by TGF-*β*, could enhance the action of TGF-*β* on cells [131]. In DM, these factors prompt cardiomyopathy fibrosis and reduced compliance of the heart [13, 20, 21]. It has been evidenced that NOX4 is the most responsible factor for ROS-induced activation of fibroblast and mesangial cells and performs an essential part in the activation of TGF-*β*1 signaling and differentiation into a profibrotic myofibroblast phenotype and matrix production [132]. This implies that antioxidant stress can also be used to fight fibrosis. Several studies have evidenced that enhancing Nrf2 could antifibrosis via inhibiting TGF-*β*. Liao et al. demonstrated that those 6 months of myricetin treatment (200 mg/kg/d) could upregulate Nrf2, HO-1, NQO1, and SOD, as well as downregulate collagen I, collagen III, fibronectin, CTGF, Smad3, and TGF-*β* [133]. Ma et al. observed that bakuchiol in vitro could upregulate Nrf2, SOD, and GPX, as well as downregulate collagen I, collagen III, *α*-smooth muscle actin (*α*-SMA), TGF-*β*, and Smad3 [134]. *α*-SMA is the biomarker of mature myofibroblasts, which is also regarded as a cardiac fibrotic marker, and the mechanism behind it might be involved in the contraction and remodeling of the extracellular matrix [135]. Ying et al. treated mice with phloretin (10 mg/kg every 2 days) for 7weeks and showed that phloretin could upregulate Nrf2, HO-1, and NQO1 and, meanwhile, downregulate TGF-*β*, collagen I, and CTGF [136].

The increased activation of the TGF-*β* signaling pathway is one of the underlying mechanisms for increased rates of apoptosis [20]. Smad3 is the chief transcription factor of TGF-*β*, and the elevation of its phosphorylation levels in human T2DM islets indicates an autocrine role for TGF-*β*/Smad3 signaling in the apoptosis of *β*-cells; the same results are observed in diabetic mice [137]. NOX 4 regulates the activation of Smad2/3 to mediate the TGF-*β*1-induced transformation of fibroblasts to myofibroblasts [132]. Increasing the expression of Nrf2 could be antiapoptotic via inhibiting TGF-*β*. Alshehri et al. gave diabetic rats a daily oral dose of kaempferol solution (50 mg/kg) and then observed that Nrf2, GSH, and Bcl2 upregulated; meanwhile, TGF-*β*1 and Bax were downregulated [138].

The main crosstalk mechanisms between Nrf2 and TGF-*β* are as follows. Firstly, Nrf2 could reduce MMP-9 to decrease the levels of TGF-*β* [139, 140]. Secondly, Nrf2-mediated Smad inhibition could be tightly associated with enhanced Smad7 levels [141]. Smad7 could form a complex type I receptor, and it recruits Smad-mediated ubiquitination regulatory factor 1/2 to activate the type I receptor, thus negatively regulating the TGF-*β* signaling pathway [141]. Zhang et al. detected that 20-week oral of notoginsenoside R1 (7.5, 15, and 30 mg/kg/d) could upregulate Nrf2, HO-1, *γ*-GCS, NQO1, and Smurf2 as well as downregulate TGF-*β*, collagen I, Bax/Bc12, caspase-3, caspase-9, and Smad2/3 [40]. Li et al. showed that 8 weeks oral of syringaresinol (25 mg/kg every other day) in diabetic mice could upregulate Nrf2, NQO1, HO-1, and SOD, as well as downregulate TGF-*β*, fibronectin, *α*- SMA, Smad2/3, Bax/Bc12, and Keap1 [142]. There exists a tight relationship between fibrosis, apoptosis, and oxidative stress, and Nrf2-dependent combating oxidative stress would be a potential therapeutic strategy.

**Figure 3:**
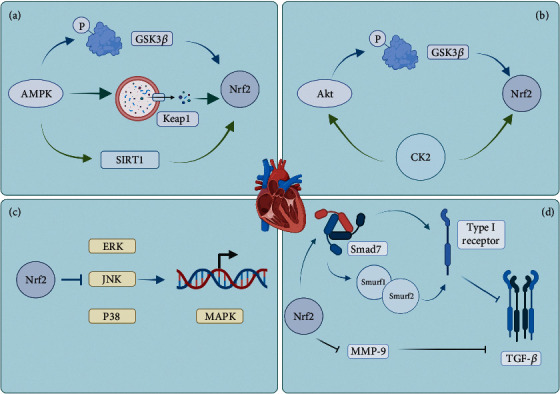
The crosstalk between Nrf2 and other signaling pathways (created with BioRender.com). (a) AMPK could phosphorylate both Nrf2 (active) and GSK3*β* (deactivate) to prevent Nrf2 ubiquitinated by GSK3*β* to improve the nuclear accumulation of Nrf2. AMPK could promote autophagic degradation of Keap1. Besides, AMPK could activate SIRT1 to regulate Nrf2. (b) Akt could phosphorylate GSK3*β* to prompt nuclear accumulation of Nrf2. Furthermore, CK2 could not only activate Akt signaling pathway but also could directly phosphorylate Nrf2 to enhance its stability. (c) ERK, JNK, and p38 are always observed accompany by suppression of Nrf2. But the underlying mechanism is unclear. (d) Nrf2 could reduce MMP-9 to decrease the levels of TGF-*β*. Besides, Smad7 could form a complex type I receptor, and it recruits Smurf 1/2 to activate the type I receptor, thus negatively regulating TGF-*β* signaling pathway. And Nrf2 could enhance the level of Smad7. AMPK: adenosine monophosphate-activated protein kinase; GSK3*β*: glycogen synthase kinase 3*β*; SIRT: silent information regulator 1; Akt: phosphatidylinositol 3-kinase-protein kinase B; CK2: ceramide-protein kinase C *ζ*-casein kinase 2; ERK: extracellular signal-regulated kinase; JNK: c-Jun N-terminal kinase; MMP: matrix metalloproteinases; TGF-*β*: transforming growth factor-*β*; Smurf: Smad-mediated ubiquitination regulatory factor.

The mechanisms of crosstalk between Nrf2 and AMPK, Akt, MAPK, and TGF-*β* signaling pathways have been summarized in the figure (see Figure 3). The above-mentioned mechanisms of Chinese herbal monomers are summarized in Table 2.

## 3. Conclusions

The increasing morbidity and lethality of DCM related to poor prognosis and survival in patients with DM call for multiple measures to prevent it. The mechanisms behind the pathogenesis of DCM are highly complex, but the overlapping progression and many signaling pathways are involved in it. What is highlighted is that oxidative stress is one of the central mechanisms in the pathogenesis of DCM. Nrf2 signaling pathway is essential to counterbalance oxidative stress via crosstalk with other signaling pathways. Surprisingly, increasing studies indicate that Chinese herbal monomers attenuate DCM in different aspects at the same time via regulating Nrf2. However, the molecular mechanisms behind the crosstalk between Nrf2 and these pathways need to be explored furthermore deeply. The phenomenon of multiple targets regulation based on Chinese herbal monomers is looking forward to having more detailed and precise experiment results.

## Figures and Tables

**Table 1:** Mechanisms of diverse signaling pathways in the pathogenesis of DCM.

Signaling pathway	Mechanism
NF-*κ*B signaling pathway	Increase inflammation and apoptosis	
AMPK signaling pathway	Improve utilization of glucose Maintain energy homeostasis Decrease lipid accumulation, ROS production, and inflammation	
Akt signaling pathway	Regulate insulin signaling Decrease apoptosis	
MAPK signaling pathway	Regulate insulin signaling Lead growth and remodeling responses Increase inflammation	
TGF-*β* signaling pathway	Increase fibrosis and apoptosis	

**Table 2:** Mechanisms behind Chinese herbal monomers ameliorate DCM based on Nrf2.

Reference	Author	Component	Experiment	Mechanism
[43]	Eman M Abdelsamia	Curcumin	In vivo	Upregulate Nrf2, HO-1
[44]	Mustafa S Atta	Thymoquinone	In vivo	Upregulate Nrf2, SOD Downregulate iNOS, NO
[47]	Guan Wang	Resveratrol	In vivo	Upregulate Nrf2, HO-1, SOD, NQO1
[48]	Z Dong	Gastrodin	In vitro	Upregulate Nrf2, SOD, CAT
[49]	Jialin Duan	*Aralia taibaiensis*	In vitro	Upregulate Nrf2, SOD, GSH
[51]	Rodrigo L Castillo	Quercetin	In vivo	Upregulate Nrf2, HO-1, SOD, PGC-1*α*
[45]	Raish Mohammad	Sinapic acid	In vivo	Upregulate GPX, SOD, CAT, I*κ*B-*α*/*β*, Nrf2, HO-1 Downregulate TNF-*α*, IL-6, NF-*κ*B
[69]	Yonggang Lian	Chrysophanol	In vivo	Upregulate Nrf2, HO-1 Downregulate IL-6, IL-18, IL-1*β*, TNF-*α*, ICAM-1, VCAM-1
[70]	Xuemei Chen	Kaempferol	In vitro	Upregulate Nrf2, HO-1, NQO1, SOD, I*κ*B-*α* Downregulate TNF-*α*, IL-6
[71]	Ershun Liang	Andrographolide	In vivo	Upregulate SOD, Nrf2, HO-1, I*κ*B-*α* Downregulate p65, NF-*κ*B, TNF-*α*, IL-1*β*, IL-6, Bax/Bc12
[72]	Hao Li	Piceatannol	In vitro	Upregulate Bc12, Nrf2, HO-1, SOD, I*κ*B-*α* Downregulate Bax, p65, caspase3
[74]	Huo Yan	Scutellarin	In vivo	Upregulate SOD, CAT, GPX, GST, Nrf2, NQO1, HO-1, I*κ*B-*β* Downregulate Keap1, TLR4, Myd88, p50, IL-6, TNF-*α*
[75]	Zhou Xu	Bixin	In vivo	Upregulate Nrf2, SOD, HO-1, CAT Downregulate TLR4, Myd88, I*κ*B-*α*, NF-*κ*B
[77]	Li Li	Luteolin	In vivo	Upregulate Nrf2, HO-1, NQO1 Downregulate IL-1*β*, IL-6, TNF-*α*, MCP-1, ICAM, VCAM
[8]	Li Ran	Bailcalin	In vivo	Upregulate AMPK*α*, SOD, CPT-1, PGC1-*α*, GSH, Nrf2 Downregulate ANP, BNP, *β*-MHC, ACC, GSSG
[93]	Fawang Du	Notoginsenoside R1	In vitro	Upregulate Nrf2, HO-1, AMPK Downregulate ANP, BNP
[95]	Ramoji Kosuru	Pterostilbene	In vivo	Upregulate SOD, CAT, GSH, GPX, PGC-1*α*, Nrf2, HO-1, AMPK Downregulate IL-1*β*, IL-6, TNF-*α*, NF-*κ*B, TLR4, NLRP3
[96]	Cuihua Zhao	Fortunellin	In vivo	Upregulate SOD, Nrf2, HO-1, AMPK Downregulate TNF-*α*, IL-1*β*, IL-6, IL-18, NF-*κ*B, Keap1
[97]	J Z Altamimi	Ellagic acid	In vivo	Upregulate GSH, SOD, Nrf2, SIRT1 Downregulate BNP, TNF-*α*, IL-6
[100]	Yiqiu Cao	Z-ligustilide	In vitro	Upregulate AMPK, Nrf2, SOD Downregulate GSK3*β*
[113]	Lijiao Xu	Scutellarin	In vivo	Upregulate Nrf2, HO-1, Akt
[110]	Sai Ma	Resveratrol	In vivo	Upregulate Nrf2, SIRT1 Downregulate ANP, BNP
[114]	Hongyang Liu	Spiraeoside	In vitro	Upregulate Akt, Nrf2, HO-1, Bcl2, SOD, GPX, CAT Downregulate caspase3, caspase7, Bax
[117]	Bin Zhang	Myricitrin	In vitro	Upregulate Nrf2, HO-1, *γ*-GCS, NQO1, Akt Downregulate GSK3*β*
[116]	Jialin Duan	Butin	In vivo	Upregulate SOD, Nrf2, HO-1, Akt Downregulate Keap1, GSK3*β*, Fyn
[118]	Xuemei Gu	Isoliquiritigenin	In vitro	Upregulate Nrf2, HO-1 Downregulate TNF-*α*, IL-6, IL-1*β*, VCAM-1, MCP-1, JNK, ERK, p38
[124]	Qitong Lu	Hinokinin	In vivo	Upregulate Nrf2, HO-1, SOD Downregulate Keap1, JNK1, ERK1/2, p38
[125]	Jing Ni	Salidroside	In vitro	Upregulate Nrf2, HO-1 Downregulate ERK, JNK, p38
[133]	Hai-Han Liao	Myricetin	In vivo	Upregulate Nrf2, HO-1, NQO1, SOD Downregulate collagen I, collagen III, fibronectin, CTGF, Smad3, TGF-*β*
[134]	Wenshuai Ma	Bakuchiol	In vitro	Upregulate Nrf2, SOD, GPX Downregulate collagen I, collagen III, *α*-SMA, TGF-*β*, Smad3
[136]	Yin Ying	Phloretin	In vivo	Upregulate Nrf2, HO-1, NQO1 Downregulate TGF-*β*, collagen I, CTGF
[138]	A S Alshehri	Kaempferol	In vivo	Upregulate Nrf2, GSH, Bcl2 Downregulate TGF-*β*1, Bax
[40]	Bin Zhang	Notoginsenoside R1	In vivo	Upregulate Nrf2, HO-1, *γ*-GCS, NQO1, Smurf2 Downregulate TGF-*β*, collagen I, Bax/Bc12, caspase-3, caspase-9, Smad2/3
[142]	Guangru Li	Syringaresinol	In vivo	Upregulate Nrf2, NQO1, HO-1, SOD Downregulate TGF-*β*, fibronectin, *α*-SMA, Smad2/3, Bax/Bc12, Keap1
